# Protein quality as a complementary functional unit in life cycle assessment (LCA)

**DOI:** 10.1007/s11367-022-02123-z

**Published:** 2022-12-28

**Authors:** G. A. McAuliffe, T. Takahashi, T. Beal, T. Huppertz, F. Leroy, J. Buttriss, A. L. Collins, A. Drewnowski, S. J. McLaren, F. Ortenzi, J. C. van der Pols, S. van Vliet, M. R. F. Lee

**Affiliations:** 1 Net Zero and Resilient Farming, Rothamsted Research, North Wyke, Okehampton, EX20 2SB Devon UK; 2 Bristol Veterinary School, University of Bristol, Langford, Bristol, BS40 5DU UK; 3 Global Alliance for Improved Nutrition (GAIN), Washington, DC 20036 USA; 4 Institute for Social, Behavioral and Economic Research, University of California, Santa Barbara, CA 93106 USA; 5 Wageningen University and Research, Wageningen, The Netherlands; 6 Dairy Physics and Chemistry, FrieslandCampina, Wolvega, Weststellingwerf The Netherlands; 7 Industrial Microbiology and Food Biotechnology (IMDO), Faculty of Sciences and Bioengineering Sciences, Vrije Universiteit Brussel, Pleinlaan 2, B-1050 Brussels, Belgium; 8 Academy of Nutrition Sciences, London, UK; 9 Department of Epidemiology, University of Washington, Nutritional Sciences, Seattle, WA 98195 USA; 10 New Zealand Life Cycle Management Centre, Massey University, Palmerston North, New Zealand; 11 Independent Nutrition and Global Health Consultant, Geneva, Switzerland; 12 Faculty of Health, School of Exercise & Nutrition Sciences, Queensland University of Technology, Brisbane, QLD 4059 Australia; 13 Center for Human Nutrition Studies, Utah State University, Logan, UT 84322 USA; 14 Harper Adams University, Edgmond, Newport, TF10 8NB UK

**Keywords:** Amino acids, Nutrition, Environmental footprints, Food, Digestibility, Health, Nutritional LCA

## Abstract

**Goal and theoretical commentary:**

A number of recent life cycle assessment (LCA) studies have concluded that animal-sourced foods should be restricted—or even avoided—within the human diet due to their relatively high environmental impacts (particularly those from ruminants) compared with other protein-rich foods (mainly protein-rich plant foods). From a nutritional point of view, however, issues such as broad nutrient bioavailability, amino acid balances, digestibility and even non-protein nutrient density (e.g., micronutrients) need to be accounted for before making such recommendations to the global population. This is especially important given the contribution of animal sourced foods to nutrient adequacy in the global South and vulnerable populations of high-income countries (e.g., children, women of reproductive age and elderly). Often, however, LCAs simplify this reality by using ‘protein’ as a functional unit in their models and basing their analyses on generic nutritional requirements. Even if a ‘nutritional functional unit’ (nFU) is utilised, it is unlikely to consider the complexities of amino acid composition and subsequent protein accretion. The discussion herein focuses on nutritional LCA (nLCA), particularly on the usefulness of nFUs such as ‘protein,’ and whether protein *quality* should be considered when adopting the nutrient as an (n)FU. Further, a novel and informative case study is provided to demonstrate the strengths and weaknesses of protein-quality adjustment.

**Case study methods:**

To complement current discussions, we present an exploratory virtual experiment to determine how Digestible Indispensable Amino Acid Scores (DIAAS) might play a role in nLCA development by correcting for amino acid quality and digestibility. DIAAS is a scoring mechanism which considers the limiting indispensable amino acids (IAAs) within an IAA balance of a given food (or meal) and provides a percentage contribution relative to recommended daily intakes for IAA and subsequent protein anabolism; for clarity, we focus only on single food items (4 × animal-based products and 4 × plant-based products) in the current case exemplar. Further, we take beef as a sensitivity analysis example (which we particularly recommend when considering IAA complementarity at the meal-level) to elucidate how various cuts of the same intermediary product *could* affect the interpretation of nLCA results of the end-product(s).

**Recommendations:**

First, we provide a list of suggestions which are intended to (a) assist with deciding whether protein-quality correction is necessary for a specific research question and (b) acknowledge additional uncertainties by providing mitigating opportunities to avoid misinterpretation (or worse, dis-interpretation) of protein-focused nLCA studies. We conclude that as relevant (primary) data availability from supply chain ‘gatekeepers’ (e.g., international agri-food distributors and processors) becomes more prevalent, detailed consideration of IAA provision of contrasting protein sources needs to be acknowledged—ideally quantitatively with DIAAS being one example—in nLCA studies utilising protein as a nFU. We also contend that future nLCA studies should discuss the complementarity of amino acid balances at the meal-level, as a minimum, rather than the product level when assessing protein metabolic responses of consumers. Additionally, a broader set of nutrients should ideally be included when evaluating “protein-rich foods” which provide nutrients that extend beyond amino acids, which is of particular importance when exploring dietary-level nLCA.

## Introduction

Actions to mitigate environmental impacts caused by human activities, including climate change, freshwater depletion and fossil fuel depletion, should be supported by a robust and quantitative estimation of the various contributing factors. One method that is commonly used to assess these impacts is life cycle assessment (LCA). Since the early Twenty-first century onwards (Heller and Keoleian 2003), LCA has been evolving into a complex tool for assessing environmental impacts and LCA researchers have begun to incorporate nutritional science into environmental LCA studies when assessing agri-food products (Heller et al. 2013). This analysis has become known as nutritional LCA (nLCA) (McAuliffe et al. 2020; McLaren et al. 2021). One of the major issues with nLCA, currently, is the use of simplified ‘nutritional functional units’ (nFUs). nFUs provide a common unit of analysis for standardising comparative nLCA of alternative food items. One of the most common ways to include nutritional functionality (i.e., the amount of food required to achieve a certain quantity of a given nutrient or nutrients) in LCA is to use an nFU of protein (e.g., Doran-Browne et al. 2015; Poore and Nemecek 2018a; Teixeira et al. 2013; Xu et al. 2018). However, (n)FUs, whether nutrition-based (e.g., calories or protein) or using other denominators such as mass (e.g., kg of product) or land occupation, either across a supply chain or a geographic area (for instance, m^2^ required to produce *x* amount of single or multiple products; McAuliffe et al. 2022a, b), have a profound effect on the interpretation of outcomes of an LCA (March et al. 2021). The choice of a nFU is therefore one of the most critical decisions in establishing the initial goal of an LCA study, regardless of any product *or* service (e.g., canteen/restaurant operations) focus.

As mentioned, nLCA has been growing in popularity and, under its current level of maturity, means it can address human health impacts derived from dietary risk factors using epidemiological data (Stylianou et al. 2016, 2021). nLCA can also consider the ramifications of changes in diet within various populations (Sonesson et al. 2017, 2019). As Sonesson et al. (2017) showed the use of protein *quantity* as a nFU, whilst benefiting from simplicity and fewer data requirements, does not address the complexities of the composition and balance of amino acids and, subsequent digestion and absorption in the human gut of each amino acid (Berrazaga et al. 2019). As a result, it is a sub-optimal metric for detailed comparisons between animal- and plant-sourced foods rich in protein despite its widespread use (Poore and Nemecek 2018a). Also, protein alone does not represent the overall nutritional value of a protein-rich food as it omits assessment of the accompanying micro- and macro-nutrient composition. These nutrients (taken as whole) vary between different food items which arguably means exploring protein in isolation (whether quality-corrected or not) is not a suitable (n)FU to represent the function of food(s); nevertheless, the fact remains that protein is one of the most widely used (n)FUs in agri-food nLCA thus warranting the current discussion (McLaren et al. 2021). In addition to the limitations of protein as an nFU, focusing on protein (or even composite nFUs for that matter) omit complexities such as anti-nutritional factors (ANFs), including but not limited, to phytates and oxalates that reduce protein digestibility (Raes et al. 2014). Some nLCA studies assess ‘nutrient density’ (i.e., a composite of nFUs; McAuliffe et al. 2018, Saarinen et al. 2017) and also include minerals, vitamins and other bioactive molecules that a consumer might expect to receive from protein-rich foods (McAuliffe et al. 2020; Lee et al. 2021a, b). However, when protein *is* adopted as an agri-food system nFU, it should, as a minimum, be contextualised according to IAA quality in order to account for variability in IAA content and composition and ultimately digestibility. As nLCA is a burgeoning field in relative infancy compared with its root framework (LCA, first conducted in the 1960s; Curran 2012), there is considerable opportunity to improve the method. Therefore, tackling issues such as protein composition and digestibility, as well as wider nutrient contents, ANFs and bioavailability, as mentioned above but not covered herein, are aspects which require attention to push the frontiers of nLCA beyond the current level of maturity through interdisciplinary collaboration. A full literature review of nLCA benefits and risks can be found in McLaren et al. (2021).

When comparing animal- and plant-sourced foods, it has recently been argued that a focus on protein overlooks the true variety of functions that are provided by foods (Leroy et al. 2022). This school of thought is particularly important in terms of how overly simplistic product labelling can misguide consumers into believing they are consuming nutritious and sustainably produced products when this may, in fact, not be the case. Indeed, there is arguably enough protein produced globally from numerous food-sources to feed the world’s population until 2030 White and Gleason 2022). However, the distribution of IAAs is currently unevenly shared between high- and low-income countries, partly due to a greater reliance on staple crops in the latter countries (Kang et al. 2021). Compared to other nutrients (e.g., fats which are amongst the most easily absorbed nutrients consumed Astrup et al., 2020), there are complexities surrounding timescales of post-prandial aminoacidemia, and therefore excess amino acids from one meal cannot always complement deficiencies in another meal (Adhikari et al. 2022; Sá et al.). As a result, this article discusses some weaknesses in the current use of nLCA from the perspective of protein digestibility and quality following consumption of different protein-based food items and their associated amino acid balances. The aim is to provide a steppingstone discussion and hypothetical case study which can be used to further the development of more insightful and nutritionally relevant nLCAs. The following section provides nutrition principles of IAAs to demonstrate the importance of acknowledging protein-based complexities. Further, a case exemplar of how to achieve improvements to protein-focused nLCA is provided under the upcoming discussion (Section 2.3).

## Indispensable amino acid quality theory and quantitative case study regarding theoretical applications in nLCAs

### Amino acid content of food items

In the most basic sense, amino acid content does not refer to *availability* but to the *amount* of indispensable and dispensable amino acids stored in the mass of a given product (e.g., mg of leucine/100 g protein). In that regard, IAAs are primarily responsible for protein anabolism, whilst dispensable amino acids can be synthesised by the body with sufficient dietary protein intake (Tipton et al. 1999; Volpi et al. 2003; NRC, 1989), though direct consumption of dispensable amino acids is important for optimal physical function and growth (Hou et al. 2015). Moreover, due to varying protein requirements at various stages of life, ratios of certain amino acids are also important. For this study, dispensable (or non-essential) amino acids will not be explored, and IAAs are the sole focus of the study in the context of nFUs and their influence on the results of protein-based nLCA studies (Table 1). For clarity, Table 1 demonstrates how different food items comprise various IAA compositions thereby setting the premise pertaining to IAA content which can subsequently be used to assess the *quality* of protein digestibility. Further, rate-limiting IAAs (i.e., those present at a low concentration in dietary protein relative to human requirements) result in suboptimal postprandial protein synthesis rates (Yang et al. 2012), if these amino acids are not provided in sufficient quantities by complementary protein sources (e.g., van Vliet et al. 2015). In humans, amongst some of the most consumed foods such as soybeans and grains e.g., Finnie and Svensson, 2014, the limiting amino acids are methionine in the case of soybeans and lysine, tryptophan and threonine in the case of grains (Brody 1999). Whilst arguably not a major issue for most people in high-income countries, the reliance on cereal-based staple crops in low-to-middle-income countries is resulting in suboptimal dietary amino acid intake for at least 1 billion people (Wu et al. 2014). Thus, interventions in such populations need to involve increasing dietary diversity and affordability and access to foods with higher contents of rate-limiting IAAs.

**Table 1:** Indispensable amino acids from major foods consumed across the globe. Source: USDA (2019)

	Product	Beef	Cheese	Eggs	Pork	Nuts	Peas	Tofu	Wheat
	USDA code^b^	23482	01009	01123	10002	12061	16085	16127	08144
IAA^a^	HIS	0.712	0.547	0.309	0.894	0.539	0.586	0.191	0.159
	ILE	0.921	1.206	0.671	1.027	0.751	0.983	0.324	0.234
	LEU	1.695	1.939	1.086	1.773	1.473	1.680	0.498	0.436
	LYS	1.859	1.025	0.912	1.947	0.568	1.771	0.431	0.219
	MET	0.553	0.547	0.380	0.577	0.157	0.195	0.084	0.138
	PHE	0.801	1.074	0.680	0.877	1.132	1.151	0.319	0.300
	THR	0.914	1.044	0.556	0.957	0.601	0.813	0.268	0.201
	TRP	0.210	0.547	0.167	0.238	0.211	0.159	0.102	0.072
	VAL	0.987	1.404	0.858	1.121	0.855	1.035	0.331	0.283

*HIS* histidine, *ILE* isoleucine, *LEU* leucine, *LYS* lysine, *MET* methionine, *PHE* phenylalanine, *THR* threonine, *TRP* tryptophan, *VAL* valine

^a^Indispensable amino acids, all values reported as g/100 g product raw

^b^Each number, or code, represents a single product within the USDA database

However, even in high-income countries, dietary protein and amino acids are of critical importance in the case of rapid growth (e.g., children), acute or chronic disease, which is particularly relevant in view of the increasing share of people with suboptimal metabolic health, and ageing populations (Bauer et al. 2013). In terms of health-related issues, Conigrave et al. (2008) found that dietary protein corresponded strongly with bone health, particularly hip fracture risk and post-injury recovery, with leucine being identified as the most important amino acid in a protective role. As nLCA grows in sophistication, it is inevitable that consideration of dietary health impacts will become more prevalent (Stylianou et al. 2021). This invariably suggests that complexities such different health status, life-stage and physical activity will eventually need to be considered within the framework. Given the existing complexity of incorporating diets of a *healthy* population into the environmental–nutrition nexus, this will add an additional layer of further work for public health nutritionists and environmental scientists. With appropriate dietary consideration, *most* humans can source all amino acids from plant-based foods as they enter the adult phase of their lives (Mariotti and Gardner 2019), provided they have no major intolerances or allergies. However, these discussions rarely go beyond ‘the possible’ and omit practicalities and issues of dietary optimisation, and plant-only diets require extra attention with respect to optimising nutritional composition compared to omnivorous, pescatarian or even vegetarian diets. This means, for example, that parents need to be more aware of nutritional composition when feeding their children solely plant-based diets (Baroni et al. 2018; Kersting et al. 2018; Parikh et al. 2021). Whether this is practical for parents on a population-based level is a topic for debate (Hoek et al. 2004) and beyond the scope of the current study. Furthermore, appropriate amounts of minimally processed animal-derived foods, when consumed along with plant-based foods, can be synergistic and thus improve the overall balance of a diet, not only for protein but for all micro- and macro-nutrients (Clegg et al. 2021).

### Digestibility

The digestibility (digestion and absorption) of protein in humans is notoriously difficult to quantify due to the necessity to carry out in vivo studies in people. To overcome this challenge, digestibility of IAAs is often measured in rats and pigs as human gut proxies (Mathai et al. 2017). Prior to the development of the Digestible Indispensable Amino Acid Score (DIAAS), digestibility was typically measured through a balance of intake and remaining amino acids in faeces in a rat assay (e.g., the protein digestibility-corrected amino acid score, or PDCAAS; Schaafsma 2000). However, questions have been raised about the representativeness of such a scoring mechanism (Mathai et al. 2017; Phillips 2017). Faecal matter can be an unreliable quantification as it contains endogenous and microbially derived amino acids and loses amino acids to colonic protein synthesis, which influence the true bioavailability of dietary amino acids (Schaafsma 2000; Marinangeli and House 2017). To overcome such issues, the DIAAS was developed and subsequently supported by the United Nations’ Food and Agricultural Organization (FAO) and the World Health Organization (WHO). The DIAAS system uses true ileal digestibility, more commonly from pigs rather than rats, as pigs are considered to have digestive tracts more similar to that of humans. Further, by utilising the gut of pigs in in vivo trials, the bioavailability of individual amino acids is better captured, making the approach more scientifically robust, although limitations remain (e.g., amino acid requirements are often assessed in fast growing pigs, whereas in adult humans amino acids are generally used for maintenance and DIAAS). Compared to PDCAAS, DIAAS is often untruncated beyond 100% as will be demonstrated in Section 2.3. The logic for untruncated scoring relates to the fact that some amino acids provide additional benefits, especially in the context of a mixed meals, where IAAs of one source can make up for the lack in other dietary protein sources (such as combining protein-rich animal foods with plant foods in a mixed meal) (Boye et al. 2012). According to Adhikari et al. (2022), DIAAS is considered the ‘ratio of IAA_lim_ in test protein compared to reference protein corrected for ileal digestibility of IAA_lim_’ (with IAA_lim_ referring to the limiting amino acid within a given food item), and the DIAAS equation can be found in Table 3 of the same paper. Complementarity of IAAs may balance out over- and under-supply amongst individual food items in the context of entire meals. Truncation on the other hand, ‘caps’ the value of protein sources (and their compositional amino acids) at 100% thereby unacknowledging their potential complementarity when consumed with other foods; in other words, if an individual product provides more than 100% of a given amino acid, or indeed a DIAAS score, it will not be credited as such. This suggests that its application is often unjustified when food items may rebalance a limiting nutrient (rate-limiting IAAs such as lysine and methionine for instance).

Not all scientists approve of DIAAS, despite being supported by the FAO (FAO 1981), to replace previous methods of calculating amino acid digestibility scores for various food items. For instance, because of the variation of nitrogen-to-protein content of many food items (which do not always align with the FAO’s assumption of 16% or a nitrogen conversion factor of 6.25), it is thought that DIAAS unfairly ‘benefits’ animal- over plant-sourced products (Craddock et al. 2021), particularly when values are untruncated. This point of view (i.e., the non-truncation of protein digestibility scores which DIAAS tends to promote) becomes particularly pertinent in the context of nLCA. In particular, foods are rarely eaten in isolation and data requirements to estimate DIAAS are often based on a single product rather than an entire meal (particularly those comprising multiple protein sources, such as eggs and beans). Moreover, such complementarities may provide various nutritional benefits beyond proteins, thereby making single commodity nLCA using protein as a nFU sub-optimal if not methodologically defunct (see Section 3 for more information). With this in mind, it is worth exploring why DIAAS was developed. Unlike meat and other animal-based products, many plant-based products contain ANFs such as lectins, phytates and tannins. These ANFs can, in certain circumstances, prevent the uptake of nutrients, including amino acids, in the human gut. However, the total food matrix is complicated, and well-planned plant-based diets contain a multitude of other compounds purported to have beneficial effects on health. These benefits may partially offset the negative effects of ANFs, in addition to ANF mitigation through food processing such as fermentation and cooking (Petroski and Minich 2020). Regarding nutritional–environmental impacts (i.e., nLCA), the question then becomes whether the protein provision of food items *needs* to be corrected for the digestibility and range of different IAAs in those food items.

### Case study and implications for nutritional life cycle assessment

nLCA takes many forms, and there has been substantial foundational research carried out in the field (e.g., see McLaren et al. 2021 for detailed information). However, to the best of the authors’ knowledge, protein quality in the form of adjusted scores based on amino acid balances and true ileal digestibility has not yet been explored in-depth in the field of nLCA. To address this knowledge gap, we carried out a virtual experiment starting from previous LCA environmental data from Poore and Nemecek (2018b). We took several commonly consumed animal- and plant-based products (Tables 1 and 2) and adjusted two environmental impact metrics: global warming potential; GWP_100_), reported as kg CO_2_-eq/100 g protein, and land use (LU), reported as m^2^*year/100 g protein. Poore and Nemecek (2018a) report impacts on a protein basis from ‘cradle to retail ready for purchasing.’ This explains why we paired environmental impacts of ‘raw’ products with their nutritional content within the aforementioned system boundary (i.e., cradle-to-retail), making Poore and Nemecek (2018a) an ideal data-source for exploratory examination. As demonstrated by the USDA’s Nutritional Database (2019; Table 1), even within single food items, the content of amino acids (and other nutrients for that matter) is determined by numerous factors including the part of the product (e.g., cut of meat, see Table 3 for a case exemplar) or processing practises. To determine the effects of quality-correction of protein on the GWP and LU metrics, we adjusted baseline functional units (i.e., impacts/100 g protein) reported by Poore and Nemecek (2018a) using best available DIAAS information from Adhikari et al. (2022; Table 2). Protein quality adjustment was carried out both without quality adjustment (i.e., a ‘control’ scenario) to represent the commonly used nFU of ‘protein’ and with untruncated DIAAS quality adjustment to illustrate how a food item’s protein content may interact with other compounds in various protein-rich foods.

**Table 2:** Digestible Indispensable Amino Acid Scores (DIAAS) and limiting indispensable amino acids (IAA_LIM_) from Adhikari et al. (2022)

Product category		Product	DIAAS (%)^a^	IAA_LIM_
Animal-sourced foods		Beef	130	Valine
		Cheese	141	Methionine
		Eggs	122	Methionine
		Pork	139	Valine
Plant-sourced foods		Nuts	86	Lysine
		Peas	88	Valine
		Tofu	105	Methionine
		Wheat	43	Lysine

^a^Where direct data for the products covered in Poore and Nemecek (2018a) were unavailable in Adhikari et al. (2022), the best available substitute was used

**Table 3:** Ratios of protein and saturated fatty acids in various cuts of beef as per the USDA SR Legacy Database (USDA 2019)

Cut	USDA code	SFA%^a^	TP%^b^	SFA: TP
Chuck	13351	5.28	26.50	0.20
Rib	13392	9.24	24.73	0.37
Top loin	13446	5.10	28.19	0.18
Porterhouse	13463	7.04	24.47	0.29
Ground (75% lean)	23577	9.59	15.76	0.61
Ground (95% lean)	23557	2.18	21.41	0.10

^a^Saturated fatty acids

^b^Protein

**Fig. 1:**
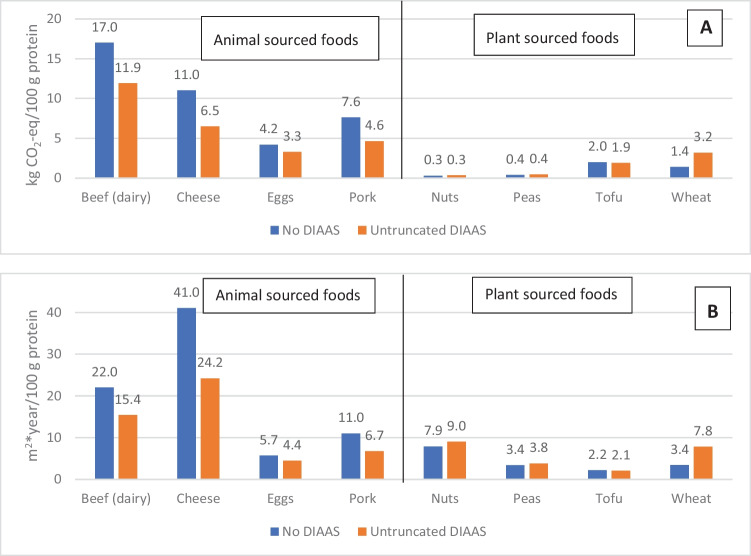
Differences in **A** global warming potential (GWP_100_; kg CO_2_-eq / 100 g protein) and **B** land use (LU; m.^2^*year) / 100 g protein) per product according to internationally weighted averages calculated by Poore and Nemecek (2018a) when products are either uncorrected for protein quality (No DIAAS) or corrected using untruncated DIAAS (as labelled in both graphs), based on DIAAS values reported in Table 2. Whilst protein values are reported in Poore and Nemecek (2018a) for most products, the value used for wheat was unidentifiable (cereals were simply reported as ‘variable protein’); as a result, we adopted the protein value from the same food commodity used in Table 1 which equated to 11.2% protein (USDA 2019; product code 08,144); however, the GWP value was transformed from bread to wheat and may therefore be slightly misaligned with primary processing into consumable wheat, though the protein content was not sensitive following a check with similar food items in USDA (2019)

Results uncorrected for quality via DIAAS demonstrate that animal-sourced products have the highest GWP (kg CO_2_-eq/100 g protein) (Fig. 1A) and LU (m^2^*year/100 g protein) (Fig. 1B**)** across all considered food items, except for eggs in the context of LU (where nuts are ranked fourth moving eggs to fifth; Fig. 1B). Untruncated DIAAS, particularly for animal-based products which tend to have high DIAAS values compared to *most* plant-protein sources (Table 2), can be quite different from the protein content values (Fig. 1B). For example, dairy beef’s GWP and LU reduce from 17 kg to 11.9 CO_2_-eq/100 g protein and from 22 to 15.4 m^2^*year/100 g protein, respectively, when comparing the results for protein content with a quality corrected DIAAS nFU. The largest change across plant-based products is wheat which, due to its low DIAAS score, results in a 57% increase in its GWP and LU impacts (Fig. 1B). Needless to say, all percentage changes in GWP and LU are driven by the DIAAS percentage reported in Table 2 as these were the coefficients used to transform the nFUs. Whilst the application of DIAAS-adjusted protein values is a useful yet simple way of considering complexities of protein accretion via individual IAAs, we would suggest that, under current data availability, the best-case scenarios available are semi-crude ‘scoping’ or ‘sensitivity’ analyses. We suggest these analyses should be reported alongside a non-adjusted protein (n)FU to take a step towards addressing uncertainties related to nutritional incorporation in LCA. The following two Sections 3 and 4 draw the reader’s attention to limitations pertaining to the proposed approach and ongoing efforts to improve its application, respectively.

## Limitations pertaining to single-nutrient functional units: protein

Development and use of nLCA is gaining traction, as demonstrated by the recent FAO publication on this method, which addresses inherent strengths and weaknesses (McLaren et al. 2021). Since protein is a common functional unit (or denominator/scaling factor of environmental impacts) used to compare various individual food items, we build upon this critical work by investigating the potential relevance of amino acid composition *and* quality when assessing protein content in nLCA. Our results, whilst simply indicative rather than definitive, suggest that using DIAAS as a correction factor (by multiplying the DIAAS % by the protein content/100 g product in the current case) has some notable effects on the quantified environmental impacts of major food items. However, these findings should be interpreted with caution. This is largely due to the fact that a person would need to eat less of a product with a high DIAAS score than a product with a low DIAAS score to achieve a well-balanced composition of digestible IAA, reinforcing the importance of consumers eating a diverse diet which levels-out over- and under-supply of protein across the different food groups. That said, our virtual experiment used pre-existing DIAAS values summarised by Adhikari et al. (2022) and substantial uncertainties remain regarding protein quality adjustment as a useful amendment for nLCA. Further research is required in this area using either, ideally, direct IAA/DIAAS values from a supply-chain under consideration (which is highly difficult to achieve, but ongoing nonetheless, due to existing data limitations despite rapid growth in this area) or more up-to-date values indirectly linked to the food systems being analysed (McAuliffe et al. 2020; Lee et al. 2021a). Regardless of the approach adopted when using protein as a functional unit, it is worth bearing in mind that protein ratios (e.g., with fat or carbohydrate, respectively) differ greatly depending on the cut of meat or section of plant being utilised (Table 3), and average values often do not represent the specific food item under consideration Franco et al. 2010; Rice et al. 2017. As a result, we recommend that a protein-content sensitivity analysis be carried out when the cut of meat or plant section being consumed by a human in a nLCA is unknown, using the lowest, average and highest protein values available to give the end-user an idea of how the protein content may affect the environmental impacts associated with the food they are consuming. This sensitivity analysis is strongly recommended especially in the absence of digestibility correction.

It is also important to reflect that amino acids sourced from proteins are only one compilation of vital nutrients contained within these food items (Leroy et al. 2022). Ideally, in future nLCAs, a wider nutrient density analysis (e.g., the NRF9.3 scoring system devised by Fulgoni et al. 2009) should be performed to more robustly align environmental impact(s) to the wider functionality of foods (i.e., to provide complete nutrition; Lee et al. 2021a). Even then, food items are not consumed in isolation but as part of a diet and, perhaps more importantly in the current context, a meal due to reasons outlined in Section 1 (i.e., the rapid uptake and excretion of IAAs/proteins). Therefore, when analysed at the single commodity level, synergies between complementary dietary ingredients, even simple combinations thereof such as rice/veg/protein source, are ignored. This is a major flaw in many nLCA and could be rectified by exploring the food matrix using omics-based approaches (also referred to as food-omics). Furthermore, carbon footprints and LU pertaining to a commodity are just two of many sustainability metrics which need to be considered together to develop a better understanding of the *holistic* sustainability of alternative products; other important impacts include—but are not limited to—eutrophication, acidification, direct and indirect land use change, animal welfare, social well-being and economic viability. Future work, including that of the current consortium, is addressing this much broader nexus of complexities and trade-offs by incorporating the nutritional sciences into nLC(S)A, with ‘S’ standing for sustainability and referring to the holistic LCA approach which covers all three pillars of sustainability: economics, environmental and social.

## Recommendations for future protein-related life cycle assessments

To reiterate, the aim of this manuscript is to provide a steppingstone discussion and hypothetical case study which can be used to further the development of more insightful and nutritionally relevant nLCAs. Although protein quality adjustment (DIAAS in the current case exemplar) can be adopted as a useful way to elucidate further nutritional information when protein is used as an (n)FU in LCA, as discussed in Section 3, it is not without its limitations. Based on this, we propose a number of considerations for LCA practitioners to decide upon before using protein as a (n)FU or indeed protein quality correction:

1. According to the goal and scope of a given study, is consideration of protein *content* (as opposed to *quality*) necessary to answer the research question? In other words, is an nLCA required, whether tier 1, tier 2, or tier 3 according to McAuliffe et al.’s (2020) proposed complexity levels, to answer a specific question to elucidate the study’s goals? If not, then mass or volume (e.g., kg grain flour or litres of milk leaving the system boundary) may be a more suitable FU. One example of not requiring nLCA is demonstrated by Lee et al. (2021b) whose work showed that beef-loin quality from three different pasture-based grazing systems did not differ notably in terms of holistic nutritional composition (including protein). Thus an nLCA would not add value in a comparison of these three systems.
2. When protein *is* deemed a necessary (n)FU (e.g., comparing protein content of dietary supplements), further consideration will undoubtedly be required to determine if the protein *quality* of such products in a comparative nLCA would lead to a different interpretation of the study’s findings; in such a case, then a quality-corrected sensitivity analysis is recommended, when both protein and protein-quality-adjusted (n)FUs should be reported side-by-side with a discussion of the differences between each FU.
3. If protein quality is being adjusted (regardless of the ‘scoring’ system), it is recommended, as far as feasibly possible, that the same data-sources be used for (a) the protein content of a food item and (b) the protein quality of said food item(s).
4. If food items provide a range of nutritional benefits (or risks) in addition to protein (for instance, legumes including soybean and peas, or animal-based products such as lean meat), then, under the nLCA framework, a composite nFU is recommended to capture the broader sustenance provided by such products (an example being Fulgoni et al.’s 2009 NRF 9.3).
5. Regardless of decision-making pertaining to 1–4 above, if an LCA practitioner is not experienced in the nutritional sciences, an appropriate collaboration should be formed with nutritional scientists to ensure that data-sources and modelling assumptions are as robust and defensible as possible.

Although these five recommendations provide a steppingstone to improve the rigour of protein focussed LCA, there are other more sophisticated steps that need to be taken in the near future. Direct primary supply–chain data is lacking when it comes to the nutrition–environment nexus; however, LCA and nutritional science research groups across the globe are beginning to forge alliances to work alongside industry to source primary data on nutrient composition of individual food items, which will enable more accurate calculations thereby negating the need to rely on secondary data. This is a time-consuming yet essential step in the evolution of nLCA, but as ‘sustainability’ becomes an ever-increasing topic in the food sector, industry actors are realising that they need to provide scientists with better quality data to truly assess the environmental footprints of their products relative to their competitors. Numerous ‘spin-out’ projects from McLaren et al. (2021) are now materialising with an end-goal of providing LCA practitioners with the aforementioned high-quality data to achieve more precise nLCAs. Additionally, these efforts also aim to address issues such as how to best compare meal-level, diet-level, and product-level nLCA including a wider range of nutrients than just protein. In conclusion, the aim of this work was to raise awareness on the limitations of nLCAs (using protein as a case study) and provide recommendations on how to improve their conduct by taking protein quality into account. As the field progresses, a broader number of nutrients provided by protein-rich foods will be explored both in terms of content *and* quality (e.g., bioavailability corrections).
